# Risk Factors for Premenopausal Breast Cancer in Bangladesh

**DOI:** 10.1155/2015/612042

**Published:** 2015-07-01

**Authors:** Javaid Iqbal, Tahmina Ferdousy, Rahela Dipi, Reza Salim, Wei Wu, Steven A. Narod, Joanne Kotsopoulos, Mohammad G. Mostafa, Ophira Ginsburg

**Affiliations:** ^1^Women's College Research Institute, Women's College Hospital, Toronto, ON, Canada M5G 1N8; ^2^Dhaka Medical College Hospital, Dhaka 1000, Bangladesh; ^3^Amader Gram Breast Care Program, Khulna 9000, Bangladesh; ^4^Bangabandhu Sheikh Mujib Medical University, Dhaka 1000, Bangladesh; ^5^Faculty of Medicine, Dalla Lana School of Public Health, University of Toronto, Toronto, ON, Canada M5T 3M7; ^6^Anowara Medical Services, Dhaka 1000, Bangladesh

## Abstract

*Background*. The incidence of premenopausal breast cancer is rising throughout South Asia. Our objective was to determine the role of risk factors associated with Westernization for premenopausal breast cancer in Bangladesh.* Methods*. We conducted a matched case-control study between January 1, 2007, and December 31, 2010, at four hospitals in Bangladesh. Cases were premenopausal women diagnosed with invasive breast cancer. Controls were premenopausal women with no personal history of breast cancer. Logistic regression was used to calculate the odds ratios (OR) for breast cancer.* Results*. We identified 129 age-matched pairs. The mean age of breast cancer diagnosis was 37.5 years. Each year decrease in the age of menarche significantly increased the risk of breast cancer (OR = 1.67, 95% CI 1.09–2.56, *P* = 0.02). The risk was also increased with a current body mass index of ≥25 kg/m^2^ (OR = 5.24, 95% CI 1.10–24.9, *P* = 0.04). Age at first childbirth, parity, and breastfeeding were not significantly associated with premenopausal breast cancer risk (*P* > 0.05).* Conclusions*. Age at menarche and adult weight gain were associated with premenopausal breast cancer risk. Other factors associated with Westernization may not be relevant to premenopausal breast cancer risk in Bangladesh.

## 1. Background

The World Health Organization (WHO) estimates that more than 60% of new cancer cases occur in low- and middle-income countries of Africa and Asia as well as Central and South America [1, 2]. These regions account for 70% of the world's total cancer deaths [2]. With increasing life expectancy and economic development, the disease burden in many low- and middle-income countries is shifting from primarily infectious diseases to chronic, noncommunicable diseases [3, 4]. Some cancer rates are predicted to increase, while others will decrease with human development [4].

The adoption of western lifestyles and changes in diet has led to an increase in the number of overweight and obese women, as well as changing reproductive patterns, such as an earlier menarche, delayed childbearing, low parity, and decreased breastfeeding [5–7]. These factors have been collectively described as “Westernization” and may have a significant impact on breast cancer risk and prognosis [8]. As a consequence, age-standardized incidence rates for breast cancer are rising in low- and middle-income countries where historically women were at a lower risk for breast cancer [9].

To date, most epidemiological studies have evaluated risk factors for breast cancer in western populations (i.e., Europe and North America). The epidemiology of breast cancer in most Asian populations is less well understood. Recent studies from several East Asian countries (e.g., China, Japan, and Republic of Korea) have shown that women in these countries increasingly share risk factors for breast cancer with women from western countries [10–12]. In comparison to East Asia, there have been far fewer studies of risk factors for breast cancer in South Asia. Most studies were conducted in India and only few evaluated the role of reproductive risk factors [13, 14] or anthropometric factors [15] according to menopausal status.

Bangladesh is a low-income country situated in South Asia, bordered by Burma, India, and the Bay of Bengal [16]. In recent decades, Bangladesh has achieved significant progress in socioeconomic development, health, and population control [17–19]. Like other South Asian countries, breast cancer is the most common malignancy among women in Bangladesh [1]. Unlike women in high-income countries, more women in South Asian countries (including Bangladesh) are diagnosed with breast cancer before menopause [20–23]. The purpose of the current study was to describe risk factors for premenopausal breast cancer in Bangladesh. To our knowledge this is the first epidemiological study of premenopausal breast cancer risk factors in Bangladesh.

## 2. Methods

### 2.1. Study Design

This multicenter, hospital-based, case-control study was conducted between January 1, 2007, and December 31, 2010, at four hospitals in Bangladesh. Of the four study sites, three are located in urban Bangladesh and include the Dhaka Medical College Hospital, Bangabandhu Sheikh Mujib Medical University, and Anowara Medical Services. The fourth study site, the Amader Gram Breast Care Program, is located in city of Khulna and mostly caters to rural patients.

#### 2.1.1. Cases

We defined a case as a premenopausal woman aged 25 years or older who was diagnosed with invasive breast cancer in the 12 months prior to study enrollment. Women were considered to be premenopausal if they had reported a menstrual period within the 12 months prior to their breast cancer diagnosis. Women who had not experienced a menstrual period for 12 consecutive months or women who had a hysterectomy were considered postmenopausal and were excluded from the study. Women who had previous history of breast cancer (*in situ* or invasive) were also not eligible for this study. Cases were recruited from the inpatient and outpatient departments of four study sites.

#### 2.1.2. Controls

A potential control was a premenopausal woman aged 25 years or older who had no prior history of breast cancer and who was unrelated to cases. Controls were recruited from outpatient clinics or women who presented at a study center in response to recruitment flyers in local community centers.

### 2.2. Data Collection

We developed a study questionnaire to record information on demographic, reproductive, and anthropometric factors. The questionnaire collected information on date of birth, income (Bangladeshi taka per month), education, age at menarche, age at first childbirth, total number of children, duration of breastfeeding (in months), current height (meters), weight at the age of 18 years (kg), and weight at the time of breast cancer diagnosis (or at interview, for controls). For each study subject we calculated body mass index (BMI) by the weight in kilograms divided by the square of the height in meters (kg/m^2^). We defined four categories of BMI according to the World Health Organization guidelines [24]. The categories were (1) underweight, <18.50 kg/m^2^, (2) normal range, 18.50 to 24.99 mg/m^2^, (3) overweight, ≥25 mg/m^2^, and (4) obese ≥30 mg/m^2^. For both cases and controls, we relied on self-reported values of weight in order to calculate the BMI at the age of 18 years. Interviews were conducted by a member of the Bangladesh-based research team.

### 2.3. Ethical Considerations

Informed consent from cases and controls was obtained by a member of the research team. The Research Ethics Boards of the University of Toronto and the Bangladesh Medical Research Council approved the study.

### 2.4. Statistical Analyses

Cases and controls were matched 1 : 1 on the date of birth, within 12 months. We performed univariable analysis to compare the baseline characteristics of cases and their matched controls. The McNemar chi-squared test was used for categorical variables and Student's *t*-test was used for continuous variables. We performed conditional logistic regression analysis to evaluate the association of age at menarche, age at first childbirth, total number of children, duration of breastfeeding, BMI, income, and education with the risk of breast cancer. Risk of breast cancer was estimated by odds ratio (OR). For all odds ratios we calculated 95% confidence intervals (CI). We performed bivariable (unadjusted) and multivariable (adjusted) analyses. A *P* value of 0.05 or less was considered statistically significant. All *P* values were two-tailed. The data was analyzed using Statistical Analysis Software (SAS) version 9.2.

## 3. Results

We identified 129 matched pairs.  Table 1 shows the reproductive, anthropometric, and socioeconomic characteristics of breast cancer cases and their matched controls. The mean age of breast cancer diagnosis in the case group was 37.5 years (range = 25–51 years). Compared to controls, breast cancer cases had a significantly earlier average age of menarche (11.5 years versus 12.0 years, *P* = 0.0002) and age at first childbirth (20.8 years versus 22.3 years, *P* = 0.006). There was no significant difference between cases and controls for mean parity (2.2 versus 2.1, *P* = 0.69) and duration of breastfeeding (55.3 months versus 52.7 months, *P* = 0.54).

Compared to controls, cases had a significantly lower mean weight at the age of 18 years (42.8 kg versus 44.8 kg, *P* = 0.009) but a significantly higher weight at the time of breast cancer diagnosis (55.7 kg versus 53.7 kg, *P* = 0.01). The average weight gain since the age of 18 years was significantly higher for cases, when compared with controls (13.0 kg versus 8.9 kg, *P* = 0.0001). Compared to controls, a significantly greater proportion of cases had a BMI in excess of ≥25 kg/m^2^ at the time of breast cancer diagnosis (cases = 27% versus controls = 7%, *P* = 0.0001).

Table 2 shows the unadjusted and adjusted OR and 95% confidence intervals for breast cancer. Age of menarche was significantly earlier in cases on univariable and multivariable analyses. From the age of 15, each year of decrease in the age of menarche was associated with almost twofold increased risk of breast cancer, when adjusted for other reproductive, anthropometric, and socioeconomic factors (OR = 1.67, 95% CI 1.09–2.56, *P* = 0.02). Age at first childbirth, parity, and breastfeeding were not associated with the risk of breast cancer.

The BMI at the age of 18 years was not associated with breast cancer risk. However, there was a trend towards increasing risk of breast cancer with weight gain since the age of 18. Each kilogram of weight gain from the age of 18 years was associated with a 7% increase in breast cancer risk (OR = 1.07, 95% CI 0.97–1.19, *P* = 0.18). Compared to women with a normal* current* BMI (18.5–24.99 kg/m^2^), the unadjusted OR of breast cancer for women with a BMI of ≥25 kg/m^2^ was 7.61 (95% CI 2.68–21.64, *P* = 0.003). The increased risk of breast cancer with a BMI of ≥25 kg/m^2^ remained significant when the model was adjusted for other factors (OR = 5.24, 95% CI 1.10–24.9, *P* = 0.04).

Lastly, in order to examine the effect of rural versus urban residence, we compared cases and controls after excluding 18 patients who were diagnosed at the rural center of Khulna. Exclusion of these patients from analyses did not substantially affect the estimates (data not shown).

## 4. Discussion

The main objective of this study was to evaluate the role of reproductive and anthropometric factors with the risk of premenopausal breast cancer in Bangladesh. Of the reproductive factors, only an early age at menarche significantly increased the risk of premenopausal breast cancer. From the age of 15 years, each year decrease in age of menarche increased the risk of breast cancer approximately twofold. Other reproductive factors such as a delayed first childbirth, low parity, and breastfeeding were not related to premenopausal breast cancer risk.

We did not observe an association between the BMI at the age of 18 years and premenopausal breast cancer risk. However, current BMI of 25 kg/m^2^ or above was associated with significantly higher risk of breast cancer.

The increased risk of premenopausal breast cancer with early menarche in our study is consistent with the literature from western populations [7, 25–27], as well as from two South Asian studies from India [13, 14]. In a meta-analysis of cohort and case-control studies, the Collaborative Group on Hormonal Factors in Breast Cancer estimated a 5% higher risk of premenopausal breast cancer with each year decrease in the age of menarche (relative risk = 1.05, 95% CI 1.04–1.06, *P* < 0.0001) [7]. Most studies included in this meta-analysis were conducted in the high-risk western populations (USA, Canada, and Europe). Only one South Asian study was included in the analysis [13]. The average age of menarche reported in the North American and European studies was approximately 13.0 years and was 13.7 years in the Indian study. In contrast, the average age of menarche for breast cancer cases in our study was two years earlier (11.5 years). This might be due to the birth cohort effect since the Indian study was published in 1991. The risk of breast cancer with each year decrease in age of menarche was also substantially higher in our study compared to the literature, both from western [25–27] and Indian populations (20–30%) [13, 14]. An earlier age of menarche in our study is consistent with a previous hospital-based study from Dhaka, Bangladesh [20]. However, this single institutional study, which did not stratify for menopausal status, reported that an earlier age of menarche was associated with a lower risk of breast cancer risk.

Most studies to date suggest that being overweight and obesity are associated with a lower risk of premenopausal breast cancer but are associated with a greater risk of postmenopausal breast cancer [28–30]. In a pooled analysis of prospective cohort studies, Brandt and colleagues estimated the effect of current BMI on pre- and postmenopausal breast cancer [28]. A BMI of 31 kg/m^2^ or higher was associated with 50% reduction in the risk of premenopausal breast cancer, compared to women who had a body mass index of 21 kg/m^2^ or less (relative risk = 0.54, 95% CI 0.34–0.85, *P* value for trend = 0.007). We observed a strong association between current BMI and premenopausal breast cancer risk in this Bangladeshi population. In our study, a current BMI of 25 kg/m^2^ or higher was associated with fivefold increase of risk of breast cancer as compared to women with a BMI of 18.5–24.9 kg/m^2^ (OR = 5.24, 95% CI 1.10–24.9, *P* = 0.04). Similar estimates were reported in the South Indian study [15]. In that study the risk of premenopausal breast cancer was associated with a BMI of >25 kg/m^2^ (OR = 1.33, 95% CI 1.05–1.69) and BMI of ≥30 kg/m^2^ (OR = 1.56, 95% CI 1.03–2.35).

Regarding early adulthood, most studies show an inverse relationship between body size at the age of 18 and the risk of breast cancer, irrespective of menopausal status [31–37]. In the Nurses' Health Study, Michels and colleagues observed a significant linear inverse trend between the BMI at the age of 18 years and the risk of premenopausal breast cancer [31]. Compared to women with BMI of 20.0–22.4 kg/m^2^ at 18 years, the risk of premenopausal breast cancer was halved among women with a BMI of 27.5 kg/m^2^ (adjusted hazard ratio = 0.57, 95% CI 0.41–0.81). Other studies have found no association [38, 39]. We found that BMI at the age of 18 was not associated with premenopausal breast cancer risk (OR per unit increase in BMI = 1.02, 95% CI 0.78–1.32, *P* = 0.91).

Weight gain in adulthood appears to be associated with an increased risk of postmenopausal breast cancer [32, 40, 41] while studies of weight gain conducted among premenopausal women have been inconsistent [35, 42–44]. In a recent analysis of the European Prospective Investigation into Cancer and Nutrition (EPIC) study, compared to women with low weight gain (quintiles 2 and 3: −0.44 to 0.36 kg per year), a high weight gain (quintile 5: 0.85–4.98 kg per year) since the age of 20 was associated with an increased risk of premenopausal breast cancer (HR = 1.37, 95% CI 1.02–1.85) [45]. However, in a report of World Cancer Research Fund, the total weight gained from the age of 18 years to current weight was associated with postmenopausal breast cancer risk but was not associated with the risk among premenopausal women [46]. In our study, every kilogram of weight gain from the age of 18 was associated with increased risk of premenopausal breast cancer, although the association did not reach statistical significance (adjusted OR = 1.07, 95% CI 0.97–1.19, *P* = 0.18). It is possible that the effects of body mass index and weight change on premenopausal breast cancer risk differ according to ethnicity [40, 47]. Larger studies, which include previously understudied ethnic groups, are warranted to explore this issue.

In the current study, delayed first childbirth and parity were not related to breast cancer risk in our study population in contrast to the existing epidemiologic evidence clearly demonstrating that childbearing is a protective factor [48, 49], and the risk of breast cancer is associated with the timing of first pregnancy [6, 49]. From the age of 20 years, each year of delay in the first pregnancy increases the risk by 5% for premenopausal women and by 3% for postmenopausal women [49]. A similar increase in premenopausal breast cancer risk in women with delayed parity and nulliparity was reported in the studies from India [13, 14].

The combined evidence from epidemiological studies suggests that breastfeeding is protective against premenopausal breast cancer. An analysis of 47 epidemiological studies estimated that each additional year of breastfeeding protects against breast cancer by a factor of 4.3% (95% CI 2.9–5.8, *P* < 0.0001) [50]. A previous study from India reported statistically nonsignificant decrease in the risk among women who ever breastfeed [14]. It may be difficult to estimate a true effect of breastfeeding in South Asian populations where most parous women breastfeed. For instance, breastfeeding is universal in Bangladesh and over 80% of women breastfeed their infants for an average duration of two years after childbirth.

Our study has several important limitations. The majority of controls in our study were recruited from the two hospital outpatient departments (DMCH and BSMMU) in Dhaka. However, a small proportion in Dhaka was recruited from the community. We cannot differentiate the source of our controls, which might affect our estimates. The sample size and number of matched pairs in this study are relatively small that may limit the precision of the effect of covariates on our primary outcome (breast cancer). Most covariates in our study were modeled as continuous variables. The small number of incident cases of breast cancer precluded us from formulating subcategories of exposures. Also, we did not evaluate other measures of central obesity such as waist circumference or waist-to-hip ratio. We relied on the recall of study subjects in order to estimate weight and body mass index at 18 years. We were also unable to collect adequate data on hormone receptor status of the breast cancers. Stratifying cases by estrogen receptor status may unmask associations, which are relevant to reproductive risk factors, as well as body mass index. Lastly, very few women from either the cases or control groups were able to report a family history of cancer (data not shown). This may be for social and/or cultural reasons, which have been suggested as a cause of underreporting of cancer family histories in other Asian studies [51].

## 5. Conclusions

Our observations suggest that overweight and obesity in adulthood and in particular weight gain during early adulthood may play an important role in the increasing risk of premenopausal breast cancer in Bangladeshi women. Further research is warranted to better understand the complex interactions between environmental, hormonal, and genetic factors in the etiology of breast cancer in Bangladesh and other South Asian populations. Meanwhile, it is important for public health and other relevant agencies in all countries to promote healthy lifestyles, including exercise and maintaining a healthy body weight in order to mitigate the rapid rise in some cancers, as well as cardiovascular disease and diabetes.

## Figures and Tables

**Table 1:** Characteristics of breast cancer cases and matched controls.

Risk factors	Characteristics	Case (*N* = 129)	Control (*N* = 129)	*P*
Reproductive factors	Age at menarche, years, mean	11.5	12.0	0.0002
	Age at first childbirth, years, mean	20.8	22.3	0.006
	Parity, mean	2.2	2.1	0.69
	Breastfeeding, months, mean	55.3	52.7	0.54

Anthropometric factors	Weight at the age of 18, kg, mean	42.8	44.8	0.009
	Current weight, kg, mean^†^	55.7	53.7	0.01
	Weight gain since the age of 18, kg, mean^††^	13.0	8.9	0.0001
	Current height, cm, mean	154.1	155.4	0.09
	BMI at the age of 18, kg/m^2^, mean	18.1	18.6	0.9
	Current BMI (kg/m^2^)^†††^			0.0001
	≤18.5	5 (4%)	4 (3%)	
	18.5–24.99	89 (69%)	116 (90%)	
	≥25	35 (27%)	9 (7%)	

Socioeconomic factors	Water source			0.5
	Tap	51 (39%)	58 (45%)	
	Tube well (untreated)	19 (15%)	14 (11%)	
	Tube well (treated)	59 (46%)	57 (44%)	
	Highest education			0.0001
	None or ≤grade 10	115 (89%)	87 (67%)	
	≥grade 11	12 (9%)	40 (31%)	
	Income (Bangladeshi taka per month), mean	3,454	4,370	0.2

BMI, body mass index.

^†^Current weight (kg): for cases, current weight was weight at the time of breast cancer diagnosis; for controls, current weight was weight at the time of interview.

^††^For cases: mean weight gain (kg) from the age of 18 years to weight at breast cancer diagnosis; for controls: mean weight gain (kg) from the age of 18 years to weight at the date of interview.

^†††^Current BMI (kg/m^2^): for cases, current BMI was BMI at the time of breast cancer diagnosis; for controls, current BMI was BMI at the time of interview.

**Table 2:** Conditional logistic regression analysis to estimate the odds (odds ratios, OR) of breast cancer.

Characteristic	Cases	Controls	Unadjusted OR			Adjusted OR		
			OR	95% CI	*P*	OR	95% CI	*P*
*Reproductive factors *								
Age at menarche, mean	11.5	12.0	1.81	1.33–2.45	0.0001	1.67	1.09–2.56	0.02
Age at first childbirth, mean	20.8	22.3	1.08	1.01–1.16	0.03	1.03	0.94–1.13	0.48
Parity, mean	2.2	2.1	0.96	0.78–1.17	0.68	1.11	0.78–1.56	0.56

*Anthropometric factors *								
Weight gain^†^, mean	13.0	8.9	1.13	1.07–1.20	0.0001	1.07	0.97–1.19	0.18
BMI at the age of 18 years, mean	18.1	18.6	1.06	0.96–1.17	0.24	1.02	0.78–1.32	0.91
Current BMI (kg/m^2^)^††^								
≤18.5	5 (4%)	4 (3%)	1.53	0.39–6.01	0.42	0.50	0.04–6.34	0.59
18.5–24.99	89 (69%)	116 (90%)	1			1		
≥25	35 (27%)	9 (7%)	7.61	2.68–21.64	0.003	5.24	1.10–24.9	0.04

*Socioeconomic factors *								
Income (Bangladeshi taka per month), mean	3,454	4,370	1.00	1.00-1.00	0.21	1.00	1.00-1.00	0.77
Education								
≥grade 11	12 (9%)	40 (31%)	1			1		
None or ≤grade 10	115 (89%)	87 (67%)	4.11	1.98–8.52	0.0001	5.26	1.54–17.99	0.008

BMI, body mass index; OR, odds ratio; CI, confidence interval.

^†^For cases: mean weight gain (kg) from the age of 18 years to weight at breast cancer diagnosis; for controls: mean weight gain (kg) from the age of 18 years to weight at the date of interview.

^††^Current BMI (kg/m^2^): for cases, current BMI was BMI at the time of breast cancer diagnosis; for controls, current BMI was BMI at the time of interview.

## References

[1] Ferlay J., Soerjomataram I., Ervik M. (2013). *GLOBOCAN 2012 v1.0, Cancer Incidence and Mortality Worldwide: IARC Cancer Base No. 11*.

[2] http://apps.who.int/bookorders/anglais/detart1.jsp?codlan=1&codcol=76&codcch=31.

[3] McCormack V. A., Boffetta P. (2011). Today's lifestyles, tomorrow's cancers: trends in lifestyle risk factors for cancer in low- and middle-income countries. *Annals of Oncology*.

[4] Bray F., Jemal A., Grey N., Ferlay J., Forman D. (2012). Global cancer transitions according to the Human Development Index (2008–2030): a population-based study. *The Lancet Oncology*.

[5] Peto J. (2001). Cancer epidemiology in the last century and the next decade. *Nature*.

[6] Clavel-Chapelon F., Gerber M. (2002). Reproductive factors and breast cancer risk. Do they differ according to age at diagnosis?. *Breast Cancer Research and Treatment*.

[7] Collaborative Group on Hormonal Factors in Breast Cancer (2012). Menarche, menopause, and breast cancer risk: individual participant meta-analysis, including 118 964 women with breast cancer from 117 epidemiological studies. *The Lancet Oncology*.

[8] Porter P. (2008). ‘Westernizing’ women's risks? Breast cancer in lower-income countries. *The New England Journal of Medicine*.

[9] Sasco A. J. (2001). Epidemiology of breast cancer: an environmental disease?. *APMIS*.

[10] Yuan J.-M., Yu M. C., Ross R. K., Gao Y.-T., Henderson B. E. (1988). Risk factors for breast cancer in Chinese women in Shanghai. *Cancer Research*.

[11] Lee H., Li J.-Y., Fan J.-H. (2014). Risk factors for breast cancer among chinese women: a 10-year nationwide multicenter cross-sectional study. *Journal of Epidemiology*.

[12] Iwasaki M., Tsugane S. (2011). Risk factors for breast cancer: epidemiological evidence from Japanese studies. *Cancer Science*.

[13] Gajalakshmi C. K., Shanta V. (1991). Risk factors for female breast cancer. A hospital-based case-control study in Madras, India. *Acta Oncologica*.

[14] Gajalakshmi V., Mathew A., Brennan P. (2009). Breastfeeding and breast cancer risk in India: a multicenter case-control study. *International Journal of Cancer*.

[15] Mathew A., Gajalakshmi V., Rajan B. (2008). Anthropometric factors and breast cancer risk among urban and rural women in South India: a multicentric case-control study. *British Journal of Cancer*.

[16] Central Intelligence Agency The World Factbook. https://www.cia.gov/library/publications/the-world-factbook/geos/bg.html.

[17] Chowdhury A. M. R., Bhuiya A., Chowdhury M. E., Rasheed S., Hussain Z., Chen L. C. (2013). The Bangladesh paradox: exceptional health achievement despite economic poverty. *The Lancet*.

[18] Ahmed S. M., Evans T. G., Standing H., Mahmud S. (2013). Harnessing pluralism for better health in Bangladesh. *The Lancet*.

[19] El Arifeen S., Christou A., Reichenbach L. (2013). Community-based approaches and partnerships: innovations in health-service delivery in Bangladesh. *The Lancet*.

[20] Jabeen S., Haque M., Islam M. J., Hossain M. Z., Begum A., Kashem M. A. (2013). Breast cancer and some epidemiological factors: a hospital based study. *Journal of Dhaka Medical College*.

[21] Rehman M., Ahsan A., Begum F., Rahman K. (2015). Epidemiology, risk factors and tumor profiles of breast cancer in Bangladeshi underprivileged women. *Gulf Journal of Oncology*.

[22] National Institute of Cancer Research and Hospital http://nicrhbd.org/images/Publication_Cancer_Registry_Report.pdf.

[23] Story H. L., Love R. R., Salim R., Roberto A. J., Krieger J. L., Ginsburg O. M. (2012). Improving outcomes from breast cancer in a low-income country: lessons from Bangladesh. *International Journal of Breast Cancer*.

[24] WHO Expert Consultation (2004). Appropriate body-mass index for Asian populations and its implications for policy and intervention strategies. *The Lancet*.

[25] Paffenbarger R. S., Kampert J. B., Chang H. G. (1980). Characteristics that predict risk of breast cancer before and after the menopause. *American Journal of Epidemiology*.

[26] Hsieh C.-C., Trichopoulos D., Katsouyanni K., Yuasa S. (1990). Age at menarche, age at menopause, height and obesity as risk factors for breast cancer: associations and interactions in an international case-control study. *International Journal of Cancer*.

[27] Colditz G. A., Rosner B. (2000). Cumulative risk of breast cancer to age 70 years according to risk factor status: data from the Nurses' health study. *The American Journal of Epidemiology*.

[28] van den Brandt P. A., Spiegelman D., Yaun S.-S. (2000). Pooled analysis of prospective cohort studies on height, weight, and breast cancer risk. *The American Journal of Epidemiology*.

[29] Albrektsen G., Heuch I., Hansen S., Kvåle G. (2005). Breast cancer risk by age at birth, time since birth and time intervals between births: exploring interaction effects. *British Journal of Cancer*.

[30] International Agency for Research on Cancer (IARC) (2002). *IARC Handbook of Cancer Prevention Volume 6: Weight Control and Physical Activity*.

[31] Michels K. B., Terry K. L., Willett W. C. (2006). Longitudinal study on the role of body size in premenopausal breast cancer. *Archives of Internal Medicine*.

[32] Research WCRFAIfC (2007). *Food, Nutrition, Physical Activity and the Prevention of Cancer: A Global Perspective*.

[33] Huang Z., Hankinson S. E., Colditz G. A. (1997). Dual effects of weight and weight gain on breast cancer risk. *Journal of the American Medical Association*.

[34] Morimoto L. M., White E., Chen Z. (2002). Obesity, body size, and risk of postmenopausal breast cancer: the women's health initiative (United States). *Cancer Causes and Control*.

[35] Brinton L. A., Swanson C. A. (1992). Height and weight at various ages and risk of breast cancer. *Annals of Epidemiology*.

[36] Le Marchand L., Kolonel L. N., Earle M. E., Mi M.-P. (1988). Body size at different periods of life and breast cancer risk. *The American Journal of Epidemiology*.

[37] Suzuki R., Iwasaki M., Inoue M. (2011). Body weight at age 20 years, subsequent weight change and breast cancer risk defined by estrogen and progesterone receptor status—the Japan public health center-based prospective study. *International Journal of Cancer*.

[38] London S. J., Colditz G. A., Stampfer M. J., Willett W. C., Rosner B., Speizer F. E. (1989). Prospective study of relative weight, height, and risk of breast cancer. *The Journal of the American Medical Association*.

[39] Feigelson H. S., Jonas C. R., Teras L. R., Thun M. J., Calle E. E. (2004). Weight gain, body mass index, hormone replacement therapy, and postmenopausal breast cancer in a large prospective study. *Cancer Epidemiology Biomarkers and Prevention*.

[40] Eliassen A. H., Colditz G. A., Rosner B., Willett W. C., Hankinson S. E. (2006). Adult weight change and risk of postmenopausal breast cancer. *The Journal of the American Medical Association*.

[41] Suzuki R., Orsini N., Saji S., Key T. J., Wolk A. (2009). Body weight and incidence of breast cancer defined by estrogen and progesterone receptor status—a meta-analysis. *International Journal of Cancer*.

[42] Peacock S. L., White E., Daling J. R., Voigt L. F., Malone K. E. (1999). Relation between obesity and breast cancer in young women. *American Journal of Epidemiology*.

[43] Weiderpass E., Braaten T., Magnusson C. (2004). A prospective study of body size in different periods of life and risk of premenopausal breast cancer. *Cancer Epidemiology Biomarkers and Prevention*.

[44] Michels K. B., Terry K. L., Eliassen A. H., Hankinson S. E., Willett W. C. (2012). Adult weight change and incidence of premenopausal breast cancer. *International Journal of Cancer*.

[45] Emaus M. J., van Gils C. H., Bakker M. F. (2014). Weight change in middle adulthood and breast cancer risk in the EPIC-PANACEA study. *International Journal of Cancer*.

[46] World Cancer Research Fund/American Institute for Cancer Research (2007). *Food, Nutrition, Physical Activity, and the Prevention of Cancer: A Global Perspective*.

[47] Renehan A. G., Tyson M., Egger M., Heller R. F., Zwahlen M. (2008). Body-mass index and incidence of cancer: a systematic review and meta-analysis of prospective observational studies. *The Lancet*.

[48] Hsieh C., Pavia M., Lambe M. (1994). Dual effect of parity on breast cancer risk. *European Journal of Cancer*.

[49] Leon D. A., Carpenter L. M., Broeders M. J. M., Gunnarskog J., Murphy M. F. G. (1995). Breast cancer in Swedish women before age 50: evidence of a dual effect of completed pregnancy. *Cancer Causes and Control*.

[50] Collaborative Group on Hormonal Factors in Breast Cancer (2002). Breast cancer and breastfeeding: collaborative reanalysis of individual data from 47 epidemiological studies in 30 countries, including 50302 women with breast cancer and 96973 women without the disease. *The Lancet*.

[51] Sharma K., Costas A., Shulman L. N., Meara J. G. (2012). A systematic review of barriers to breast cancer care in developing countries resulting in delayed patient presentation. *Journal of Oncology*.

